# Angiogenesis related genes *NOS3*, *CD14*, *MMP3* and *IL4R* are associated to VEGF gene expression and circulating levels in healthy adults

**DOI:** 10.1186/s12881-015-0234-6

**Published:** 2015-10-05

**Authors:** Abdelsalam Saleh, Maria G. Stathopoulou, Sébastien Dadé, Ndeye Coumba Ndiaye, Mohsen Azimi-Nezhad, Helena Murray, Christine Masson, John Lamont, Peter Fitzgerald, Sophie Visvikis-Siest

**Affiliations:** 1 UMR INSERM U 1122, IGE-PCV “Interactions Gène-Environnement en Physiopathologie Cardio Vasculaire”, Université de Lorraine, Nancy, F-54000 France; 2 Department of Medical Genetics, School of Medicine, Mashhad University of Medical Sciences, Mashhad, Iran; 3 Randox Laboratories, Crumlin, UK; 4 Geriatric Service, University Hospital of Nancy, Nancy, France

**Keywords:** VEGF, NOS3, CD14, MMP3, IL4R

## Abstract

**Background:**

Vascular endothelial growth factor (VEGF) plays a key role in angiogenesis. The aim was to assess the genetic connections between the angiogenesis-related *NOS3, CD14, MMP3, IL4R*, *IL4* genes and VEGF expression and plasma levels.

**Methods:**

The associations between VEGF plasma levels with the polymorphisms of *NOS3*, *CD14*, *MMP3*, *IL4R*, and *IL4* were assessed in 403 healthy unrelated adults. The epistatic and environmental interactions were explored, including four VEGF-related polymorphisms previously identified. The VEGF expression in peripheral blood mononuclear cells was quantified (*n* = 65) for the VEGF121, VEGF145, VEGF165, and VEGF189 isoforms.

**Results:**

The polymorphism rs1799983 of *NOS3* was associated with the sum of all VEGF isoforms mRNA levels (*P* = 0.032) and VEGF145 (*P* = 0.033). Rs1800779 of *NOS3* interacted with rs3918226 of the same gene and with the rs2569190 of *CD14* (*P* = 0.022, *P* = 0.042, respectively) for VEGF plasma levels. Other epistatic interactions included the rs1801275 of *IL4R* with the rs6921438 (VEGF-related variant) and rs3025058 of *MMP3* (*P* = 0.042, *P* = 0.010 respectively) and the rs2569190 of *CD14* with the rs3025058 of *MMP3* (*P* = 0.0119). We also identified an interaction of rs1800779 with obesity, high-density lipoprotein cholesterol and triglycerides (*P* = 0.018, *P* = 0.005, *P* = 0.043, respectively) as well as the interaction of rs6921438 with hypertension (*P* = 0.028).

**Conclusions:**

Our findings indicated that genetic variants of *NOS3*, *CD14*, *MMP3* and *IL4R* are implicated in the determination of VEGF expression and plasma levels. Thus, they support the hypothesis that in physiological conditions there are complex biological relationships between pathways (such as angiogenesis and inflammation), which are involved in the development of chronic diseases.

**Electronic supplementary material:**

The online version of this article (doi:10.1186/s12881-015-0234-6) contains supplementary material, which is available to authorized users.

## Background

Angiogenesis is the procedure of development of new vessels from the existing vasculature. One of the most potent angiogenesis regulators is the vascular endothelial growth factor A (VEGF-A or more commonly known as VEGF). VEGF is a highly conserved, disulfide-bonded dimeric glycoprotein of 34–46 kDa. It is generated by several cell types including neutrophils, macrophages, fibroblasts, peripheral blood mononuclear cells (PBMCs) and endothelial cells. Elevated circulating VEGF levels have been associated with several types of cancer and other chronic diseases including cardiovascular diseases (ischemic heart disease, heart failure, stroke), diabetes, immune and inflammatory disorders [1–3].

Other regulators of angiogenesis include the nitric oxide synthase (NOS_3_), the CD14^+^ monocytes, the matrix metalloproteinases (MMPs), and the interleukin 4 (IL4).

NOS3 provides continuous local production of nitric oxide (NO). NO is an important angiogenesis mediator and/or effector also involved in endothelial function and thereby can influence vascular tonicity, insulin resistance, development of atherosclerosis and type 2 diabetes [4, 5]. The increase in NO production via up regulation of NOS_3_ by VEGF indicates that the angiogenic effect of VEGF seems to be mediated by NO [6–8]

CD14^+^ monocytes are abundant in human peripheral blood and have strong potential to differentiate into endothelial cells [9]. CD14 is a multifunctional receptor and contributes to different biological and pathophysiological processes including apoptosis, sepsis, inflammatory diseases, angiogenesis and tumor growth. Therefore, CD14 has been demonstrated to be a candidate for developing anti-cancer medications in tumor growth and angiogenesis [10].

The MMPs are zinc-dependent proteinases with an ability to degrade enzymes [11]. They are involved in pathologic conditions characterized by excessive degradation of extracellular matrix, such as tumor metastasis, and arteriosclerosis [12]. Several pieces of evidence indicate associations between MMPs and VEGF and a link of MMPs with angiogenesis [12–18].

The IL4 is a glycoprotein secreted by activated T lymphocytes, basophils and mast cells [19]. Previous studies have indicated the involvement of IL4 in angiogenesis [20, 21].

These molecules are implicated in a variety of biological procedures in both health and disease. The precise mechanisms involving these molecules in angiogenesis in different conditions are under investigation. However, due to their complexity, interpreting the origin of their biological connections is challenging. Therefore, their evaluation in the healthy state is believed to provide important information concerning the physiological relationships between these molecules before the development of a specific disease. For this reason, the current study was performed in a supposed healthy population, aiming to examine possible genetic links between angiogenesis related (*NOS3, CD14, MMP3*, and *IL4*) candidate genes and VEGF plasma levels and gene expression in the healthy state. This assessment could provide evidence of functionally angiogenesis-related polymorphisms. Furthermore, gene-gene (epistatic) and gene-environment interactions effects on VEGF plasma levels were also investigated. Those interaction analyses included 4 polymorphisms significantly associated with VEGF plasma levels in a previous genome-wide association study (GWAS), and explaining up to 50 % of its inter-individual variability [3]. *In silico* structural analyses have been performed for the functional validation of the results.

## Methods

### Study population

The STANISLAS Family Study is a 10-year longitudinal survey involving 1,006 families from Vandoeuvre-lès-Nancy, France between 1993–1995 [22]. Exclusion criteria included the presence of chronic disorders (cardiovascular diseases or cancer) and the personal history of cardiovascular disease. The study protocol was approved by the Local Ethics Committee of Nancy and all subjects gave written informed consent. Four hundred and three unrelated adults collected during the second examination of the STANISLAS Family Study were selected based on complete availability of data.

### Data collection and biological measurements

The procedures of data collection have been previously described [22, 23]. Blood samples were collected after overnight fasting. Sodium EDTA-plasma was separated by centrifugation at 2000 g for 15 min at 4 °C and stored at −196 °C in liquid nitrogen until analysis. VEGF plasma levels were quantified by Randox Ltd (Crumlin, UK) using a biochip array analyser (Evidence ®) [24]. Total cholesterol (TC) was measured using a cholesterol oxidase-paraaminophenazone method, triglycerides using a glycerophosphate oxidase/paraaminophenazone alanylglycine glycine method, and high-density lipoprotein cholesterol (HDL-C) levels using a phosphotungstate method, while low-density lipoprotein cholesterol (LDL-C) levels were calculated using the Friedewald formula [25].

Hypertension was defined as systolic blood pressure ≥140 mm/Hg or diastolic blood pressure ≥90 mm/Hg. Body mass index (BMI) was calculated as weight (kilograms) divided by height (meters) squared. Obesity was defined as BMI ≥30 kg/m^2^. Smokers were identified based on current smoking status.

### SNP selection

The single nucleotide polymorphisms (SNPs) rs2569190, rs2243250, rs2010963, rs3918226, rs1799983, rs1800779, rs3025058, rs1805015 and rs1801275 of the *NOS3, CD14, MMP3, IL4R,* and *IL4* genes (Table 1) were selected on the basis of their functionality on the related genes.

**Table 1:** Characteristics of the studied polymorphisms

Chromosome	SNPs	Minor allele	Common allele	Closed to/on gene	Minor allele Frequency	HWE ^a^*P*
5	rs2569190	G	A	CD14 molecule	0.48	0.8332
5	rs2243250	T	C	IL4 interleukin 4	0.15	0.6865
6	rs4416670	C	C	MGC45491 and MRPL14 (near VEGF)	0.47	0.5489
6	rs2010963	C	G	VEGFA	0.35	0.3243
6	rs6921438	A	G	MGC45491 and MRPL14 (near VEGF)	0.4	1
7	rs3918226	T	C	NOS3 nitric oxide synthase 3	0.09	0.3379
7	rs1799983	T	G	NOS3 nitric oxide synthase 3	0.35	0.7309
7	rs1800779	G	A	NOS3 nitric oxide synthase 3	0.4	0.6624
8	rs6993770	T	A	ZFPM2	0.28	0.9019
9	rs10738760	G	A	VLDLR and KCNV2	0.48	0.9205
11	rs3025058	-	T	MMP3 matrix metallopeptidase 3	0.48	1
16	rs1805015	C	T	IL4R interleukin 4 receptor	0.13	0.3821
16	rs1801275	G	A	IL4R interleukin 4 receptor	0.18	0.8632

^a^Hardy Weinberg Equilibrium

The SNPs rs6921438, rs4416670, rs6993770, and rs10738760, previously shown to be associated with VEGF levels [3], were used for the testing of epistatic effects.

### DNA isolation and genotyping

The genomic DNA was isolated using the salting out method [26]. The polymorphisms rs2569190, rs2243250, rs2010963, rs3918226, rs1799983, rs1800779, rs3025058, rs1805015 and rs1801275 of the *NOS3, CD14, MMP3, IL4R* and *IL4* genes were genotyped using multilocus genotyping assays previously described [27, 28]. The VEGF-related SNPs were genotyped by Genoscreen (http://genoscreen.fr), using a Sequenom iPLEX Gold assay–Medium Throughput Genotyping Technology [29].

### Gene expression assays

Total RNA was extracted from PBMCs in a subsample of 65 subjects with a MagNaPure automate, using the MagNA Pure LC RNA HP isolation kit and RNA HP Blood External lysis protocol [Roche Diagnostics, France]. Reverse transcription of total RNAs were performed using 200 units of M-MuLV Reverse Transcriptase with 0.25 μg of oligos (dT) (Promega, France) as previously described [30]. Quantification of the transcripts coding for the VEGF isoforms (VEGF_121_, VEGF_145_, VEGF_165_, VEGF_189_), the beta 2 microglobulin (β2M) control gene were performed using TaqMan® and LightCycler technologies (LC TaqMan Master Kit, Roche Diagnostics, France) in duplicate. RT-PCR optimization and specificity of Real Time-PCR products were conducted using SYBR® Green technology (LC FastStart DNA MasterPLUS SYBR Green I kit, Roche Diagnostic, France), melting curves analysis and agarose gel electrophoresis of the PCR amplicons [30]. All mRNA levels were normalized to the mRNA levels of β2M gene. Total VEGF mRNA derived from the sum of the ratio of the four isoforms present in PBMCs.

### Computational structural analyses

**Fig. 1:**
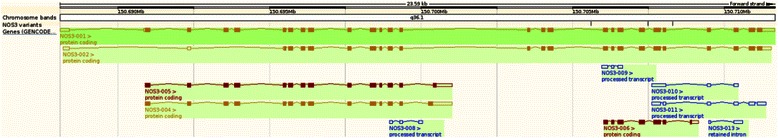
Schematic organisation of eNOS3 transcripts in chromosome 7. Rs1799983 is indicated with the orange triangle. Exons are represented by rectangular shapes and coding exons are coloured

The polymorphism rs1799983 allows the generation of two isoforms of endothelial NOS3: eNOS3 Glu298 (allele G) and eNOS3 Asp298 (allele T). This gene is located on chromosome 7 q36.1. It contains 26 coding exons extending on approximately 24 kb. Multiple transcript variants encode different isoforms (Fig. 1). Canonical isoform codes for an enzyme of 1203 amino acids. The active protein is a homodimer (chains A and B) catalyzing from L-arginine the formation of NO. The polymorphism rs1799983 is located on the coding exon 7 of *NOS3* and has been previously identified by Yoshimura et al. as a Glu298-to-Asp variant [31].

Sofowora et al. found that eNOS3 Asp298 homozygotes excreted significantly less nitrate/nitrite than eNOS3 Glu298 homozygotes [32]. As amino acid in position 298 does not belong to the catalytic residue of the enzyme active site (formed by Arg187 (A) – Cys184 (A) – Glu361 (A) – Trp356 (A)) a question remains: how this rs1799983G > T can affect nitric oxide production and how can this be linked with VEGF?

Structural analyses were performed using the crystallographic structure of the human endothelial nitric oxide synthase, oxygenase domain [33]. Conformational changes in nitric oxide synthases induced by chlorzoxazone and nitroindazoles were assessed through crystallographic and computational analyses of inhibitor potency [33]. Computational analyses for the building of a model of NOS3 Asp298 were performed using the I-Tasser suite [34, 35] and Pymol software was used to analyze structural features (the PyMol Molecular Graphics System, Schrodinger, LLC).

### Statistical analyses

VEGF plasma concentrations and mRNA values were log-transformed, before the analyses, to normalize their distribution. Hardy-Weinberg equilibrium was tested using the chi-square test.

The associations between the SNPs of *NOS3, CD14, MMP3, IL4R* and *IL4* genes and VEGF plasma and expression levels were assessed through linear regression adjusted for age, gender and BMI under an additive model and using the minor allele as reference allele.

The epistatic interactions between the polymorphisms of *NOS3, CD14, MMP3, IL4R* and *IL4* genes and the VEGF-related SNPs for VEGF plasma levels were tested using the linear models described before with the introduction of the interaction term of a 2 × 2 combination between the assessed SNPs. For example VEGF ~ age + gender + BMI + SNP1 + SNP2 + SNP1 × SNP2. The environmental factors used for the SNPs × environment interactions included smoking, blood lipids levels, obesity, and hypertension. We used linear regression models adjusted for age, gender, BMI (when obesity was the assessed factor, adjustments were performed only for age and gender), the environmental factor, and the additional interaction term (SNP × environmental factors). The interaction analyses were not performed for *VEGF* gene expression data due to the limited sample size. Analyses were performed using PLINK 1.07 (http://pngu.mgh.harvard.edu/purcell/plink) [36] and the SPSS 17.0 (SPSS, Inc, Chicago, Illinois) statistical softwares. Significance was determined at a two-tailed *P* = 0.05 level.

The sample size calculation was performed using the software QUANTO. Based on published information about allele frequencies of the selected polymorphisms, the levels of VEGF in plasma and its expression, and an estimation of a mild effect size, a number of 400 individuals would be sufficient in order to achieve a statistical power of 80 %.

## Results

The studied polymorphisms are presented in Table 1. All SNPs were in agreement with Hardy-Weinberg equilibrium (*P* > 0.05). The characteristics of the studied population are summarized in Table 2 (Additional file 1: Tables S1 and S2).

**Table 2:** Characteristics of study population (*n* = 403)

Variable	Mean^a^	SD^b^
Age (years)	44.4	4.8
Gender (%) male	50.4	
Body mass index (kg/m^2^)	24.9	3.9
Vascular endothelial growth factor (pg/ml)	42.7	43.3
Total cholesterol (mmol/l)	5.7	1.0
Triglycerides (mmol/l)	1.3	1.2
High- density lipoprotein (mmol/l)	1.6	0.4
Low-density lipoprotein (mmol/l)	3.5	0.8
Obesity (%)	7.9 %	
Smoking (%)	25.4 %	
Hypertension (%)	5.7 %	

^a^Mean values for continuous variables and percentages for categorical variables

^b^SD standard deviation

### Association of the assessed polymorphisms of the NOS3, CD14, MMP3, IL4R and IL4 genes with VEGF gene expression and plasma levels

After adjustment for age, gender and BMI, a significant association was observed between rs1799983 of *NOS3* and the sum of all VEGF isoforms mRNA and VEGF_145_ mRNA (*β* = 0.19, *P* = 0.032 and *β* = 0.17, *P* = 0.033 respectively). None of the polymorphisms of the *NOS3, CD14, MMP3, IL4R* and *IL4* genes were associated with VEGF plasma levels.

### Epistatic interactions between NOS3, CD14, MMP3, IL4R, IL4 and VEGF-related polymorphisms on the plasma levels of VEGF

Six genetic variants were involved in epistatic interactions for VEGF plasma levels regulation, including rs1800779 and rs3918226 (*NOS3*), rs2569190 (*CD14*), rs3025058 (*MMP3*), rs1801275 (*IL4R*) and rs6921438 (*VEGF*) (Table 3). Specifically, the interaction of rs1800779 of *NOS3* with rs3918226 of the same gene was associated with increased VEGF plasma levels (*β* = 0.17, *P* = 0.022) and the interaction with the rs2569190 of *CD14* (*β* = −0.06, *P* = 0.042) was associated with decreased VEGF plasma levels. Therefore, the SNP rs1800779 of *NOS3* has a modifiable effect on VEGF plasma levels though epistatic interactions with different polymorphisms. Additionally the rs1801275 of *IL4R* interacted with the VEGF-related SNP rs6921438 resulting in an increase of VEGF plasma levels (*β* = 0.07, *P* = 0.042) and with the rs3025058 of *MMP3* with a decreasing effect (*β* = −0.09, *P* = 0.010). Again, in this case, the rs1801275 has different effects on VEGF plasma levels depending on interactions with different polymorphisms of angiogenesis-related genes. Finally, the interaction between rs2569190 of *CD14* and rs3025058 of *MMP3* was associated with a significant increase in plasma levels of VEGF (*β* = 0.07, *P* = 0.019).

**Table 3:** Epistatic interactions for VEGF plasma levels

SNP × SNP	Genes	*β* ^a^(pg/ml)	*t-values*	*P*
rs1800779 × rs2569190	*NOS3* × *CD14*	−0.07	−2.04	0.042
rs1800779 × rs3918226	*NOS3* × *NOS3*	+0.17	2.29	0.022
rs1801275 × rs6921438	*IL4R* × *VEGF*	+0.08	2.043	0.042
rs1801275 × rs3025058	*IL4R* × *MMP3*	−0.10	−2.58	0.010
rs2569190 × rs3025058	*CD14* × *MMP3*	+0.07	2.36	0.019

^a^ β, effect size

### SNPs × environmental interactions on the plasma levels of VEGF

Significant interactions were observed between *NOS3* SNPs and HDL-C, triglycerides and obesity (Table 4). A decrease of VEGF plasma levels resulted from the interaction of rs1800779 with obesity and triglycerides (*β* = −0.11, *P* = 0.018, *β* = −0.04, *P* = 0.043 respectively), while its interaction with HDL-C was associated with an increase in VEGF plasma levels (*β* = 0.12, *P* = 0.005). Furthermore, the VEGF-related polymorphism rs6921438 interacted with hypertension to decrease VEGF levels (*β* = −0.18, *P* = 0.028).

**Table 4:** Significant gene x environment interactions with VEGF plasma levels

Interaction	Gene	β ^a^(pg/ml)	*t-values*	*P*
rs1800779 × HDL-C	NOS3	0.12	2.78	0.005
rs1800779 × Triglycerides	NOS3	−0.04	−1.98	0.043
rs1800779 × Obesity	NOS3	−0.11	−2.36	0.018
rs6921438 × Hypertention	MGC45491 and MRPL14(near *VEGF*)	−0.18	−2.20	0.028

^a^ β, effect size

### Structural analyses of NOS3 gene

**Fig. 2:**
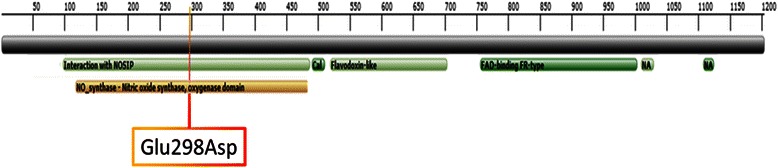
Domains’ organisation of human eNOS3 enzyme. Position of rs1799983 is indicated (Glu298Asp). NA: NADP-nucleotide phosphate-binding region, Cal: calmodulin-binding-region

The study of NOS3 domains revealed that the amino acid in position 298 is located in NOS-Interacting Protein (NOSIP) binding domain (amino acids 98–498). Moreover, this binding domain overlaps the oxygenase-domain containing active site (Fig. 2).

**Fig. 3:**
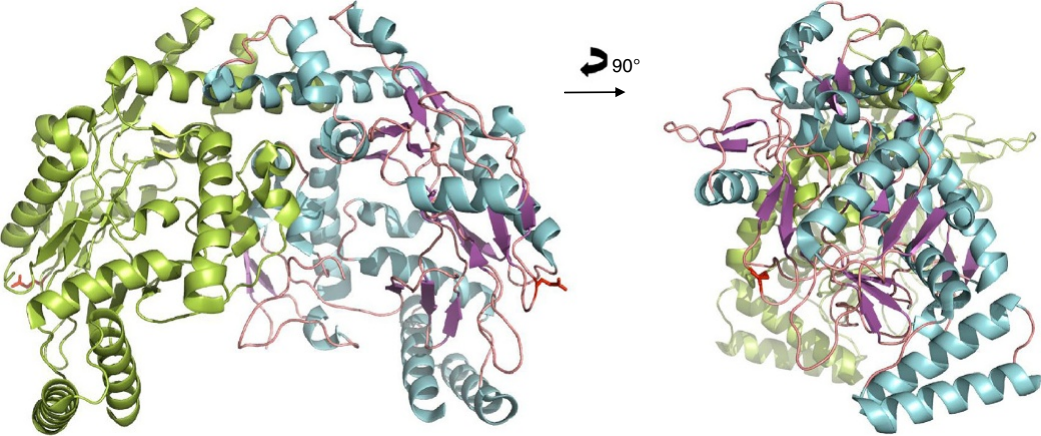
Representation of the dimeric form of eNOS3 oxygenase domains (PDB : 1M9M). Chain A is in green and chain B is represented in cyan for alpha helix and magenta for beta strands. Glu298Asp corresponding to rs1799983, is depicted in red in each chain

**Fig. 4:**
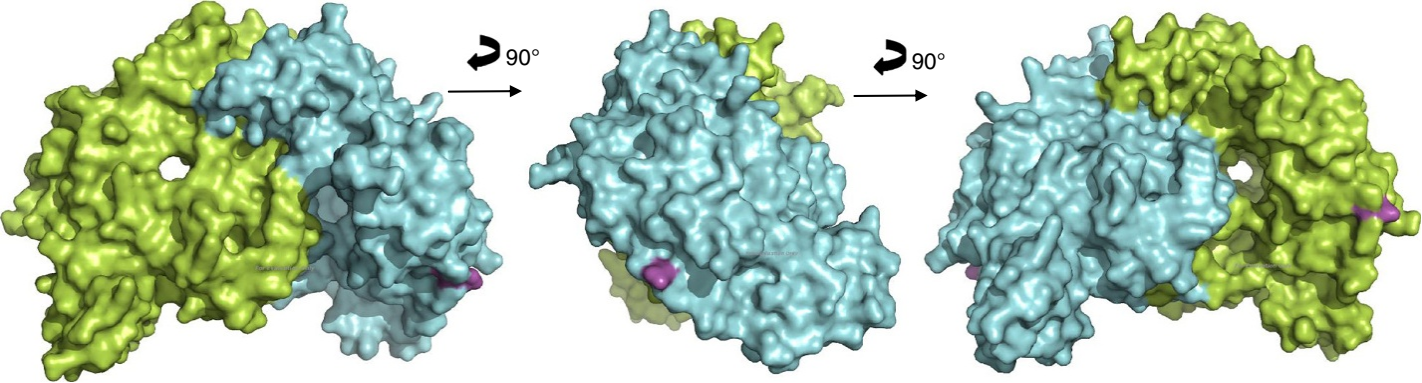
Representation of solvent-accessible surface area of eNOS3 homodimer oxygenase domains (PDB :1M9M). Chain A and B are in green and cyan respectively. Glu298 is coloured in magenta in both chains

**Fig. 5:**
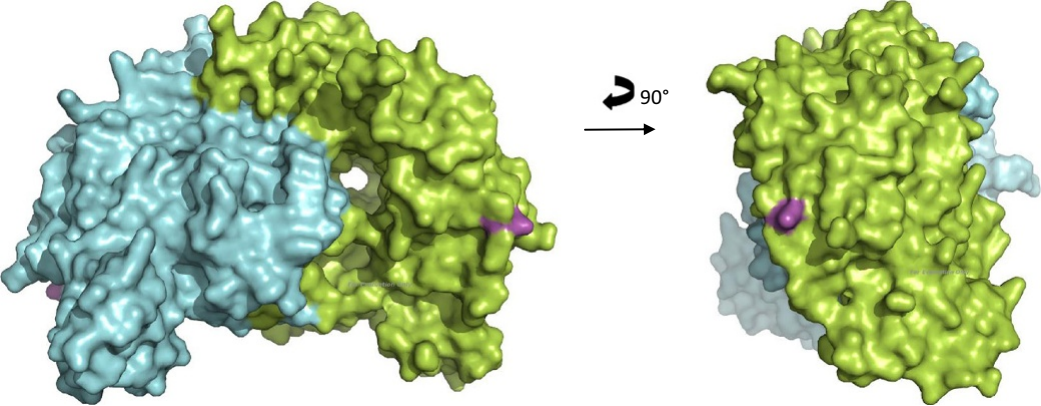
NOS3 *Glu298* [1m9m]

**Fig. 6:**
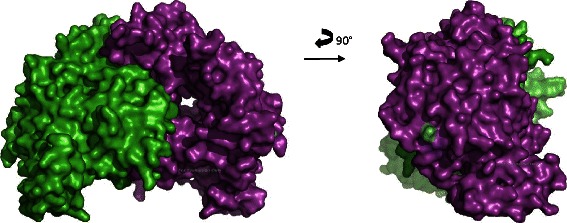
Homodimer NOS3 *Asp98* (cyan)

Comparative analyses of the crystal structure of the human eNOS3 oxygenase domain (pubmedcode :1M9M −1.96 Å resolution – Figs. 3 and 4) with the computed eNOS3 Asp298 reveals conformational changes between eNOS3 Glu298 and eNOS3 Asp298. Indeed, the amino acid in position 298 is not buried but exposed on the surface. Thereby, eNOS3 shows a protuberance when it is composed of Glu298 whereas eNOS3 model removes this relief with aspartate amino acid. (Figs. 5 and 6).

## Discussion

In the present study, we have identified significant associations between genetic variants of *NOS3* with *VEGF* gene expression in a healthy population. Furthermore, significant epistatic and gene × environment interactions have been identified for VEGF plasma levels involving polymorphisms of *NOS3, CD14, MMP3, IL4R* and VEGF-related SNPs.

Numerous SNPs have been identified in *NOS3* gene, among them, the common variant, G984T (rs1799983). It is located in exon 7 of the gene, and it modifies its coding sequence, thus resulting in an amino acidic substitution of glutamic acid to aspartic acid at position 298 of the protein. It has been reported to alter its enzymatic activity [37, 38]. This SNP has been demonstrated by several groups to be linked with the risk for coronary spasm, coronary artery disease and acute myocardial infarction [39–43].

In this study, a significant association between the SNP rs1799983 and *VEGF* expression level (total mRNA and VEGF_145_ isoform) was observed. The minor allele of this SNP was associated with increased levels of VEGF mRNA. Such association might suggest the existence of transcriptional regulation of *VEGF* from *NOS3* in PBMCs in non-pathological situations and thus a novel molecular link between VEGF and NO. It is noteworthy that in a previous investigation, we had identified significant associations between the VEGF_145_ isoform with cellular adhesion and inflammation molecules expression (*ICAM-1*, *L-selectin* and *TNF-a*) in the same population [44]. Our current results further support the importance of this isoform on cardiovascular physiology and underline its potent role as a key molecule in different diseases.

There are few pathways leading to the synthesis of NO. One of them consists in the formation of the complex eNOS3/caveolin-1. However, a protein avidly binding to the eNOS oxygenase domain termed NOSIP [45] has been identified. When NOSIP binds to eNOS3, eNOS is sequestered in the Golgi apparatus decreasing NO production in eNOS3 Asp298 homozygotes [32]. It is therefore likely that eNOS3 298 Asp promotes formation of a complex with NOSIP. In this context, homozygotes eNOS3 298 Asp probably synthesize NO via alternative pathways involving VEGF/PI3K/Akt [46]. Thus, to compensate this engagement to this preferred pathway of complex formation with NOSIP for homozygotes eNOS3 Asp298, the physiological response to NO production is the increase of *VEGF* expression levels, known to stimulate the synthesis of NO via eNOS3 on the one hand but also via VEGF/PI3K/Akt. Therefore, our structural analyses offer an explanation of the link between the SNP rs1799983 and *VEGF* expression level.

On the other hand, no significant relations were found between *VEGF* gene expression and/or plasma levels and *CD14, MMP3, IL4R and IL4* polymorphisms in our healthy population.

In this study, we also observed an epistatic interaction between *NOS3* SNP rs1800779 and *CD14* SNP rs2569190 which resulted in a significant decrease in VEGF plasma level. The rs1800779 is located in the upstream and promoter region of the gene and may eventually lead to reduced production and bioavailability of NO. Also, the *CD14* SNP rs2569190 is situated at position −159 of the gene and has been suggested to be linked with increased expression and transcriptional activity of CD14 [47].

Additionally, we have found that the interaction between the *NOS3* SNPs rs1800779 and rs3918226, was associated with a significant increase in VEGF plasma level. Similar to the former SNP, the rs3918226 is located in the promoter region of *NOS3* gene. Although no functional studies have been reported so far for this SNP, few studies have identified a transcription-factor binding site for the ETS-family next to rs3918226, suggesting that rs3918226 can potentially modulate the expression of *NOS3* [48].

It is important to mention that the epistatic interaction between *IL4R* SNP rs1801275 and the VEGF-related SNP rs6921438 was associated with a significant increase in VEGF plasma level in our healthy population. IL4 biological actions are mediated by its binding to the receptors IL4, which leads to the activation of intracellular signaling pathways [49]. The *IL4R* SNP rs1801275 is a substitution of Arg with Gln at the position 576 of the protein, resulting in an enhancement in the receptor signaling. In addition, the VEGF-related SNP rs6921438 is an intergenic variant that is located at 171 kb downstream of the *VEGF* gene on chromosome 6p21.1. In a previous GWAS, the minor allele of the polymorphism was associated with decreased VEGF levels [3]. The mechanism of this interaction is not known and as few data are available in the literature, the biological explication of this epistatic effect cannot be hypothesized.

Complex relationships have been observed between IL4 and VEGF in several diseases. IL4 has pro-angiogenic and pro-inflammatory properties in the lungs during exposure to chronic hypoxia, an effect dependent on the hypoxia-mediated induction of the VEGF signaling pathway [50]. Also, IL4 has been discovered to exert anti-angiogenic properties in several cancer models with high VEGF expression levels [51]. Moreover, in patients with rheumatoid arthritis, IL4 inhibits VEGF production in synovial fibroblasts [7]. The results of our study support the concept of crosstalk between VEGF and IL4.

Another finding of our investigation indicates an epistatic interaction between the *IL4R* SNP rs1801275 and *MMP3* SNP rs3025058 that was associated with a significant decrease in VEGF plasma level. The *MMP3* SNP is located in the promoter region of the gene where the one allele has a run of six adenosines (6A) and another having five adenosines (5A) at position 1171 [52]. It has been shown that MMPs are involved in different steps of angiogenesis and play an important role in the blood vessel growth and the process of vascular development in cancer [53]. In addition, MMPs can increase the bioavailability of VEGF [54]. The association between MMPs and VEGF has been demonstrated, in several studies, to contribute to different pathological conditions, *e.g.* cancer invasion [15–17]. Therefore, considering the potential interactions of VEGF with both IL4 and MMPs, it is not surprising that in this study we observed a significant interaction between IL4R and MMPs for VEGF plasma levels.

Hypertension, smoking, blood lipids and obesity were the “environmental factors” selected for interaction analyses, as they are risk factors with large environmental impact for different diseases, especially cardiovascular diseases. We observed interactions between *NOS3* SNP rs1800779 with HDL-C, triglycerides and obesity. These interactions were associated with increased VEGF plasma levels in the case of HDL-C, and decreased VEGF levels for triglycerides and obesity. Loebig et al. reported a positive correlation between plasma VEGF concentrations in overweight subjects as compared with normal and low weight individuals. However, they did not find any significant differences of VEGF levels when comparing normal and low weight participants [6]. Sandhofer et al., found that the plasma levels of VEGF are positively and negatively correlated with BMI in men, and visceral fat in women respectively [55]. Our interactions results indicate that the relationship between VEGF and adiposity could be mediated by *NOS3* variants, through interactions with the adiposity indexes.

Finally, we identified an interaction of the SNP rs6921438 with hypertension, which was associated with a decrease in VEGF levels. As previously mentioned, the minor allele of this variant was associated with decreased VEGF levels [3] and explained 41 % of the VEGF inter-individual variability. Furthermore, it was the only polymorphism among the 4 VEGF-related assessed in the present study that was implicated in epistatic and environmental interactions for VEGF. Therefore, it seems that its role as a VEGF biomarker may be even more significant through indirect interactions as well. Moreover, the same variant has been also recently found to contribute to decreased HDL-C and increased LDL-C levels in the same population [56]. Thus, it could represent a strong biological link between angiogenesis, blood pressure regulation and blood.

The findings of this study are original and they are providing initial evidences on the field of functional variants implicated in angiogenesis regulation, through direct effects or interactions, in health. A limitation of the study is the use of nominal significance threshold for the production of the results. This is the most common practice in the candidate genes studies, where a limited number of SNPs are assessed, like the present one. Understanding this limitation, we have performed structural analyses and provide functional biological evidences for some of these results. Furthermore, we acknowledge the need for replication of our results in larger population.

## Conclusions

In the present work, we assessed the associations of VEGF plasma levels and gene expression with *NOS3, CD14, MMP3, IL4R, IL4* and VEGF-related SNPs in a healthy population, as well as their epistatic and environmental interactions. The significant role of a functional *NOS3* polymorphism on *VEGF* gene expression was presented and biological confirmation was provided though structural analyses of *NOS3* gene. VEGF plasma levels were also affected by epistatic and environmental interactions with polymorphisms of the *NOS3*, *CD14*, *MMP3*, and *IL4R*. Among the VEGF-related polymorphisms, the stronger VEGF determinant, rs6921438, was also implicated in such interactions modulating VEGF levels. These results indicate the central role of VEGF regulation in different physiological biological procedures and support the existence of complex relationships between angiogenesis, *NOS3, CD14, MMP3 and IL4R* that exist even in the healthy state. Such complex interactions should be taken into account in future studies concerning the implication of VEGF in related human chronic pathologies. The understanding of these associations may promote the knowledge of the molecular mechanisms and the processes that mediate complex diseases such as diabetes, cancer and cardiovascular diseases. Also the possible development of new therapeutic targets in a personalized medicine approach can also be considered. Furthermore, this study underlines the value of candidate gene approach in combination with GWAS in assessing interactions of the biological determinants of disease intermediate phenotypes such as the VEGF.

## Additional file

Additional file 1: Table S1. VEGF plasma and expression levels per genotype. **Table S2.** VEGF plasma and expression levels per interaction condition. (DOCX 72 kb)

## Abbreviations

VEGF: Vascular endothelial growth factor
NOS3: Nitric Oxide Synthase
NOSIP: NOS-Interacting Protein
CD14: Cluster of differentiation
MMP3: Matrix metalloproteinase-3
IL4: Interleukin-4
IL4R: Interleukin-4 receptor
GWAS: Genome-wide association study
TC: Total cholesterol
HDL-C: High-density lipoprotein cholesterol
LDL-C: Low-density lipoprotein cholesterol
BMI: Body mass index
PBMC: Peripheral blood mononucleated cell
SNPs: Single-nucleotide polymorphism
ICAM: InterCellular Adhesion Molecule
TNFα: Tumor necrosis factor
