# Supplementary material for: Angiogenesis related genes NOS3, CD14, MMP3 and IL4R are associated to VEGF gene expression and circulating levels in healthy adults
Source: BMC Med Genet. 2015 Oct 5;16:90. doi: 10.1186/s12881-015-0234-6 (PMC4594922; doi:10.1186/s12881-015-0234-6)
Supplement: Additional file 1: Table S1. — VEGF plasma and expression levels per genotype. Table S2. VEGF plasma and expression levels per interaction condition. (DOCX 72 kb) [file 12881_2015_234_MOESM1_ESM.docx]

**Supplementary tables**

**Supp. Table 1** VEGF plasma and expression levels per genotype

| rs2243250 | | N | Mean | SD^a^ |
| --- | --- | --- | --- | --- |
| CC | VEGF plasma levels (pg/ml) | 258 | 41.47 | 34.70 |
|  | VEGF121 | 41 | 51.28 | 23.18 |
|  | VEGF145 | 33 | 52.34 | 30.44 |
|  | VEGF165 | 44 | 265.83 | 145.92 |
|  | VEGF189 | 32 | 15.14 | 7.26 |
| CT | VEGF plasma levels (pg/ml) | 96 | 40.70 | 40.60 |
|  | VEGF121 | 14 | 43.07 | 14.90 |
|  | VEGF145 | 13 | 45.22 | 16.76 |
|  | VEGF165 | 14 | 221.43 | 93.96 |
|  | VEGF189 | 13 | 13.90 | 7.36 |
| TT | VEGF plasma levels (pg/ml) | 7 | 38.19 | 35.53 |
|  | VEGF121 | 1 | 54.19 | . |
|  | VEGF145 | 1 | 19.16 | . |
|  | VEGF165 | 1 | 329.22 | . |
|  | VEGF189 | 1 | 20.95 | . |
| rs2569190 | | N | Mean | SD ^a^ |
| AA | VEGF plasma levels (pg/ml) | 96 | 44.87 | 43.84 |
|  | VEGF121 | 17 | 50.23 | 25.10 |
|  | VEGF145 | 12 | 57.85 | 35.35 |
|  | VEGF165 | 17 | 231.95 | 88.89 |
|  | VEGF189 | 11 | 15.16 | 5.20 |
| GA | VEGF plasma levels (pg/ml) | 178 | 42.23 | 36.32 |
|  | VEGF121 | 23 | 49.81 | 22.73 |
|  | VEGF145 | 21 | 51.70 | 24.68 |
|  | VEGF165 | 26 | 281.19 | 176.86 |
|  | VEGF189 | 21 | 15.07 | 9.25 |
| GG | VEGF plasma levels (pg/ml) | 87 | 35.05 | 24.78 |
|  | VEGF121 | 16 | 47.50 | 15.48 |
|  | VEGF145 | 14 | 39.59 | 21.84 |
|  | VEGF165 | 16 | 241.99 | 89.01 |
|  | VEGF189 | 14 | 14.49 | 5.18 |
| rs4416670 | | N | Mean | SD ^a^ |
| CC | VEGF plasma levels (pg/ml) | 94 | 39.42 | 61.73 |
|  | VEGF121 | 9 | 48.97 | 19.39 |
|  | VEGF145 | 7 | 36.97 | 13.63 |
|  | VEGF165 | 10 | 274.94 | 115.17 |
|  | VEGF189 | 7 | 18.05 | 5.22 |
| CT | VEGF plasma levels (pg/ml) | 194 | 40.83 | 32.34 |
|  | VEGF121 | 33 | 46.20 | 20.90 |
|  | VEGF145 | 28 | 56.36 | 28.17 |
|  | VEGF165 | 36 | 248.29 | 151.41 |
|  | VEGF189 | 27 | 13.51 | 7.07 |
| TT | VEGF plasma levels (pg/ml) | 113 | 48.98 | 41.36 |
|  | VEGF121 | 15 | 59.06 | 24.15 |
|  | VEGF145 | 13 | 41.19 | 26.95 |
|  | VEGF165 | 14 | 274.96 | 106.82 |
|  | VEGF189 | 13 | 16.54 | 7.87 |
| rs6921438 | | N | Mean | SD ^a^ |
| AA | VEGF plasma levels (pg/ml) | 79 | 21.20 | 11.74 |
|  | VEGF121 | 12 | 47.53 | 18.63 |
|  | VEGF145 | 12 | 47.68 | 23.07 |
|  | VEGF165 | 12 | 251.40 | 97.99 |
|  | VEGF189 | 12 | 16.40 | 5.04 |
| AG | VEGF plasma levels (pg/ml) | 198 | 40.29 | 31.07 |
|  | VEGF121 | 27 | 45.66 | 21.59 |
|  | VEGF145 | 22 | 46.33 | 30.55 |
|  | VEGF165 | 28 | 253.65 | 153.18 |
|  | VEGF189 | 21 | 13.07 | 7.56 |
| GG | VEGF plasma levels (pg/ml) | 123 | 60.89 | 62.34 |
|  | VEGF121 | 17 | 59.30 | 23.41 |
|  | VEGF145 | 13 | 54.54 | 25.85 |
|  | VEGF165 | 19 | 274.91 | 135.57 |
|  | VEGF189 | 13 | 16.80 | 8.14 |
| rs2010963 | | N | Mean | SD ^a^ |
|  |  |  |  |  |
| CC | VEGF plasma levels (pg/ml) | 35 | 42.81 | 31.67 |
|  | VEGF121 | 6 | 67.06 | 22.90 |
|  | VEGF145 | 6 | 58.28 | 52.07 |
|  | VEGF165 | 6 | 339.66 | 129.87 |
|  | VEGF189 | 5 | 20.38 | 9.33 |
| CG | VEGF plasma levels (pg/ml) | 153 | 39.61 | 31.26 |
|  | VEGF121 | 17 | 52.81 | 15.59 |
|  | VEGF145 | 15 | 41.79 | 17.50 |
|  | VEGF165 | 19 | 290.48 | 106.69 |
|  | VEGF189 | 15 | 15.38 | 5.33 |
| GG | VEGF plasma levels (pg/ml) | 127 | 37.43 | 30.53 |
|  | VEGF121 | 18 | 43.94 | 19.62 |
|  | VEGF145 | 16 | 45.16 | 24.61 |
|  | VEGF165 | 19 | 233.78 | 179.29 |
|  | VEGF189 | 16 | 13.82 | 8.26 |
| rs1800779 | | N | Mean | SD ^a^ |
|  |  |  |  |  |
| AA | VEGF plasma levels (pg/ml) | 127 | 39.62 | 38.72 |
|  | VEGF121 | 17 | 52.86 | 26.76 |
|  | VEGF145 | 14 | 49.20 | 28.16 |
|  | VEGF165 | 18 | 250.55 | 120.76 |
|  | VEGF189 | 14 | 15.51 | 6.95 |
| AG | VEGF plasma levels (pg/ml) | 178 | 42.44 | 33.66 |
|  | VEGF121 | 33 | 48.38 | 17.38 |
|  | VEGF145 | 29 | 50.05 | 28.41 |
|  | VEGF165 | 33 | 237.61 | 89.09 |
|  | VEGF189 | 28 | 13.88 | 6.94 |
| GG | VEGF plasma levels (pg/ml) | 56 | 40.87 | 39.00 |
|  | VEGF121 | 6 | 44.07 | 26.75 |
|  | VEGF145 | 4 | 48.44 | 22.48 |
|  | VEGF165 | 8 | 346.86 | 261.06 |
|  | VEGF189 | 4 | 20.12 | 9.41 |
| rs1799983 | | N | Mean | SD ^a^ |
|  |  |  |  |  |
| GG | VEGF plasma levels (pg/ml) | 147 | 42.20 | 38.63 |
|  | VEGF121 | 20 | 52.60 | 26.02 |
|  | VEGF145 | 14 | 37.03 | 14.76 |
|  | VEGF165 | 20 | 221.51 | 105.81 |
|  | VEGF189 | 14 | 13.53 | 7.11 |
| GT | VEGF plasma levels (pg/ml) | 170 | 38.68 | 32.38 |
|  | VEGF121 | 28 | 44.67 | 18.08 |
|  | VEGF145 | 26 | 59.05 | 31.78 |
|  | VEGF165 | 32 | 268.60 | 156.53 |
|  | VEGF189 | 25 | 15.05 | 7.63 |
| TT | VEGF plasma levels (pg/ml) | 44 | 47.63 | 41.94 |
|  | VEGF121 | 8 | 57.09 | 17.41 |
|  | VEGF145 | 7 | 40.04 | 14.06 |
|  | VEGF165 | 7 | 300.07 | 83.61 |
|  | VEGF189 | 7 | 17.20 | 6.05 |
| rs3918226 | | N | Mean | SD ^a^ |
|  |  |  |  |  |
| CC | VEGF plasma levels (pg/ml) | 298 | 41.21 | 37.33 |
|  | VEGF121 | 45 | 51.26 | 22.00 |
|  | VEGF145 | 38 | 47.96 | 23.48 |
|  | VEGF165 | 49 | 267.39 | 139.60 |
|  | VEGF189 | 38 | 15.55 | 7.55 |
| CT | VEGF plasma levels (pg/ml) | 64 | 40.82 | 30.97 |
|  | VEGF121 | 11 | 41.20 | 17.08 |
|  | VEGF145 | 9 | 56.83 | 41.04 |
|  | VEGF165 | 10 | 202.38 | 97.80 |
|  | VEGF189 | 8 | 11.90 | 4.42 |
| TT | VEGF plasma levels (pg/ml) | 1 | 35.61 | . |
|  | VEGF121 | 0 |  |  |
|  | VEGF145 | 0 |  |  |
|  | VEGF165 | 0 |  |  |
|  | VEGF189 | 0 |  |  |
| rs6993770 | | N | Mean | SD ^a^ |
|  |  |  |  |  |
| AA | VEGF plasma levels (pg/ml) | 204 | 45.00 | 49.21 |
|  | VEGF121 | 31 | 52.26 | 20.61 |
|  | VEGF145 | 23 | 52.07 | 29.53 |
|  | VEGF165 | 32 | 273.90 | 140.04 |
|  | VEGF189 | 22 | 16.28 | 6.84 |
| TA | VEGF plasma levels (pg/ml) | 164 | 40.20 | 33.71 |
|  | VEGF121 | 19 | 43.06 | 19.41 |
|  | VEGF145 | 19 | 39.92 | 21.35 |
|  | VEGF165 | 20 | 241.89 | 141.95 |
|  | VEGF189 | 19 | 14.19 | 7.26 |
| TT | VEGF plasma levels (pg/ml) | 31 | 36.38 | 39.69 |
|  | VEGF121 | 6 | 48.87 | 16.46 |
|  | VEGF145 | 6 | 69.37 | 24.17 |
|  | VEGF165 | 7 | 237.34 | 110.37 |
|  | VEGF189 | 6 | 13.09 | 8.48 |
| rs10738760 | | N | Mean | SD ^a^ |
|  |  |  |  |  |
| AA | VEGF plasma levels (pg/ml) | 97 | 54.36 | 66.19 |
|  | VEGF121 | 11 | 43.85 | 23.73 |
|  | VEGF145 | 11 | 56.21 | 40.54 |
|  | VEGF165 | 12 | 243.16 | 133.42 |
|  | VEGF189 | 10 | 16.09 | 6.10 |
| AG | VEGF plasma levels (pg/ml) | 199 | 39.41 | 30.71 |
|  | VEGF121 | 34 | 48.46 | 20.10 |
|  | VEGF145 | 27 | 46.27 | 23.05 |
|  | VEGF165 | 34 | 232.76 | 114.54 |
|  | VEGF189 | 27 | 13.66 | 8.03 |
| GG | VEGF plasma levels (pg/ml) | 104 | 36.99 | 31.32 |
|  | VEGF121 | 10 | 56.14 | 14.98 |
|  | VEGF145 | 9 | 48.08 | 19.44 |
|  | VEGF165 | 12 | 353.09 | 169.14 |
|  | VEGF189 | 9 | 17.76 | 5.25 |
| rs3025058 | | N | Mean | SD ^a^ |
|  |  |  |  |  |
| DD | VEGF plasma levels (pg/ml) | 96 | 37.02 | 28.69 |
|  | VEGF121 | 12 | 51.13 | 17.39 |
|  | VEGF145 | 12 | 40.64 | 20.06 |
|  | VEGF165 | 14 | 285.52 | 92.32 |
|  | VEGF189 | 12 | 16.75 | 6.05 |
| DI | VEGF plasma levels (pg/ml) | 182 | 41.58 | 36.15 |
|  | VEGF121 | 33 | 49.01 | 22.45 |
|  | VEGF145 | 28 | 51.70 | 24.08 |
|  | VEGF165 | 34 | 258.99 | 148.84 |
|  | VEGF189 | 28 | 14.64 | 6.86 |
| II | VEGF plasma levels (pg/ml) | 85 | 44.81 | 43.19 |
|  | VEGF121 | 11 | 48.08 | 23.66 |
|  | VEGF145 | 7 | 56.96 | 46.37 |
|  | VEGF165 | 11 | 211.16 | 134.26 |
|  | VEGF189 | 6 | 12.55 | 10.82 |
| rs1805015 | | N | Mean | SD ^a^ |
|  |  |  |  |  |
| CC | VEGF plasma levels (pg/ml) | 9 | 47.45 | 44.27 |
|  | VEGF121 | 1 | 28.04 | . |
|  | VEGF145 | 1 | 38.08 | . |
|  | VEGF165 | 1 | 174.61 | . |
|  | VEGF189 | 1 | 7.47 | . |
| TC | VEGF plasma levels (pg/ml) | 83 | 44.08 | 33.89 |
|  | VEGF121 | 12 | 51.42 | 18.62 |
|  | VEGF145 | 10 | 41.16 | 24.74 |
|  | VEGF165 | 14 | 294.70 | 174.83 |
|  | VEGF189 | 10 | 17.53 | 8.69 |
| TT | VEGF plasma levels (pg/ml) | 269 | 40.11 | 36.77 |
|  | VEGF121 | 43 | 49.18 | 22.25 |
|  | VEGF145 | 36 | 52.34 | 28.19 |
|  | VEGF165 | 44 | 246.03 | 120.79 |
|  | VEGF189 | 35 | 14.38 | 6.69 |
| rs1801275 | | N | Mean | SD ^a^ |
|  |  |  |  |  |
| AA | VEGF plasma levels (pg/ml) | 239 | 39.08 | 33.44 |
|  | VEGF121 | 40 | 50.00 | 22.39 |
|  | VEGF145 | 34 | 50.65 | 27.44 |
|  | VEGF165 | 41 | 254.64 | 119.97 |
|  | VEGF189 | 33 | 14.42 | 6.90 |
| AG | VEGF plasma levels (pg/ml) | 109 | 44.87 | 41.50 |
|  | VEGF121 | 14 | 48.28 | 19.68 |
|  | VEGF145 | 12 | 47.81 | 29.20 |
|  | VEGF165 | 16 | 267.80 | 177.23 |
|  | VEGF189 | 12 | 16.90 | 8.00 |
| GG | VEGF plasma levels (pg/ml) | 13 | 49.47 | 38.79 |
|  | VEGF121 | 2 | 41.95 | 19.66 |
|  | VEGF145 | 1 | 38.08 | . |
|  | VEGF165 | 2 | 200.46 | 36.55 |
|  | VEGF189 | 1 | 7.47 | . |

^a^SD standard deviation

**Supp. Table 2** VEGF plasma and expression levels per interaction condition.

| hypertension | | N | Mean | SD ^a^ |
| --- | --- | --- | --- | --- |
| no | VEGF plasma levels (pg/ml) | 380 | 42.40 | 43.05 |
|  | VEGF121 | 55 | 50.27 | 22.26 |
|  | VEGF145 | 46 | 49.72 | 26.98 |
|  | VEGF165 | 58 | 260.48 | 137.36 |
|  | VEGF189 | 45 | 15.18 | 7.21 |
| yes | VEGF plasma levels (pg/ml) | 23 | 48.53 | 48.47 |
|  | VEGF121 | 2 | 43.08 | 4.53 |
|  | VEGF145 | 2 | 42.58 | 41.51 |
|  | VEGF165 | 2 | 214.73 | 27.62 |
|  | VEGF189 | 2 | 11.60 | 7.30 |
| obesity | | N | Mean | SD ^a^ |
| normal | VEGF plasma levels (pg/ml) | 235 | 43.53 | 48.03 |
|  | VEGF121 | 35 | 48.63 | 23.45 |
|  | VEGF145 | 32 | 51.12 | 28.57 |
|  | VEGF165 | 35 | 251.73 | 117.36 |
|  | VEGF189 | 31 | 14.51 | 8.31 |
| overweight | VEGF plasma levels (pg/ml) | 136 | 43.18 | 38.54 |
|  | VEGF121 | 18 | 52.53 | 20.87 |
|  | VEGF145 | 13 | 42.84 | 25.62 |
|  | VEGF165 | 20 | 255.55 | 129.91 |
|  | VEGF189 | 13 | 15.67 | 4.53 |
| obese | VEGF plasma levels (pg/ml) | 32 | 35.21 | 20.05 |
|  | VEGF121 | 4 | 50.86 | 13.93 |
|  | VEGF145 | 3 | 59.87 | 14.35 |
|  | VEGF165 | 5 | 323.15 | 259.19 |
|  | VEGF189 | 3 | 17.60 | 2.57 |
| smoking | | N | Mean | SD ^a^ |
| no | VEGF plasma levels (pg/ml) | 181 | 40.90 | 36.74 |
|  | VEGF121 | 30 | 50.22 | 23.64 |
|  | VEGF145 | 26 | 46.43 | 25.08 |
|  | VEGF165 | 32 | 261.60 | 140.68 |
|  | VEGF189 | 26 | 15.26 | 7.59 |
| yes | VEGF plasma levels (pg/ml) | 102 | 43.73 | 41.33 |
|  | VEGF121 | 15 | 48.50 | 19.72 |
|  | VEGF145 | 11 | 48.64 | 21.71 |
|  | VEGF165 | 14 | 244.22 | 109.64 |
|  | VEGF189 | 11 | 15.68 | 7.48 |
| ex-smoker | VEGF plasma levels (pg/ml) | 119 | 40.61 | 28.79 |
|  | VEGF121 | 12 | 51.42 | 21.65 |
|  | VEGF145 | 11 | 57.27 | 36.36 |
|  | VEGF165 | 14 | 267.64 | 153.48 |
|  | VEGF189 | 10 | 13.69 | 6.15 |

^a^SD standard deviation
